# Is There a Role for Elective Early Upper Gastrointestinal Contrast Study in Neurologically Impaired Children following Laparoscopic Nissen Fundoplication?

**DOI:** 10.3390/children8090813

**Published:** 2021-09-16

**Authors:** Thomas M. Benkoe, Katrin Rezkalla, Lukas Wisgrill, Martin L. Metzelder

**Affiliations:** 1 Department of Pediatric and Adolescent Surgery, Medical University of Vienna, 1090 Vienna, Austria; katrin.rezkalla@meduniwien.ac.at (K.R.); martin.metzelder@meduniwien.ac.at (M.L.M.); 2 Department of Pediatrics and Adolescent Medicine, Division of Neonatology, Pediatric Intensive Care & Neuropediatrics, Medical University of Vienna, 1090 Vienna, Austria; lukas.wisgrill@meduniwien.ac.at

**Keywords:** Nissen fundoplication, laparoscopy, neurologically impaired, children, upper gastrointestinal contrast study

## Abstract

Assessment of discomfort as a sign for early postoperative complications in neurologically impaired (NI) children is challenging. The necessity of early routine upper gastrointestinal (UGI) contrast studies following laparoscopic Nissen fundoplication in NI children is unclear. We aimed to evaluate the role of scheduled UGI contrast studies to identify early postoperative complications following laparoscopic Nissen fundoplication in NI children. Data for laparoscopic Nissen fundoplications performed in NI children between January 2004 and June 2021 were reviewed. A total of 103 patients were included, with 60 of these being boys. Mean age at initial operation was 6.51 (0.11–18.41) years. Mean body weight was 16.22 (3.3–62.5) kg. Mean duration of follow up was 4.15 (0.01–16.65 years) years. Thirteen redo fundoplications (12.5%) were performed during the follow up period; eleven had one redo and two had 2 redos. Elective postoperative UGI contrast studies were performed in 94 patients (91%). Early postoperative UGI contrast studies were able to identify only one complication: an intrathoracal wrap herniation on postoperative day five, necessitating a reoperation on day six. The use of early UGI contrast imaging following pediatric laparoscopic Nissen fundoplication is not necessary as it does not identify a significant number of acute postoperative complications requiring re-intervention.

## 1. Introduction

Neurologically impaired children (NI) often experience feeding problems due to esophageal dysmotility, reduced lower esophageal pressure, increased intra-abdominal pressure, and delayed gastric emptying [1]. Several studies have demonstrated the high incidence of gastroesophageal reflux (GER) in NI children with clinical symptoms such as vomiting and regurgitation in 20–30% of this population [2]. Indications for anti-reflux surgery are symptoms or complications of gastroesophageal reflux disease (GERD) not sufficiently relieved with conservative treatment [3,4]. Nissen fundoplication has emerged as a feasible therapeutic option to treat refractory GERD in both neurological normal and NI children. In the past, laparoscopic Nissen fundoplication has gained acceptance and shown to be as effective and safe as open Nissen fundoplication. Despite its common use in pediatric surgery and decades of performance, high rates of complications and recurrences after Nissen fundoplication in NI patients have been reported, even if these results are not homogenous [5,6].

The recognition and treatment of complications in the acute post-operative period remains to be elucidated. The early treatment of complications is often more straightforward than if left undiagnosed [7,8]. Moreover, early interventions do not necessarily prolong post-operative recovery and may prevent difficult redo-surgery at a later stage.

The need for routine post-operative contrast imaging following anti-reflux surgery is currently questioned in adult surgery. While some units prefer the use of routine postoperative contrast studies with water soluble swallows, others challenge the prognostic value of such imaging studies, bringing increased costs and prolonged hospital stay to the discussion. There is growing evidence in adult surgery that postoperative contrast studies can be performed selectively if the patients become symptomatic [9,10].

Particularly in NI children, discomfort or location of pain may be difficult to assess. Hence, the evaluation of early postoperative outcome following laparoscopic Nissen fundoplication in this delicate patient collective is challenging. Up to date there are no existing recommendations concerning the diagnostic value of early routine postoperative imaging after pediatric laparoscopic fundoplication. This is especially true for children with a neurological impairment.

The main objective of this study was to examine whether routine early contrast imaging is necessary to identify early post-operative complications requiring re-intervention following Nissen fundoplication in neurologically impaired children. 

Beside analyzing demographics like gender, age and underlying neurological impairment, we furthermore aimed to evaluate whether a pre-existing gastrostomy or simultaneous placement of a gastrostomy was associated with an increased incidence of redo-fundoplications in NI children. 

## 2. Materials and Methods

After obtaining institutional review board approval (EK 1528/2015), we performed a retrospective chart review of neurologically impaired children from the Department of Pediatric and Adolescent Surgery at the Medical University of Vienna who underwent a laparoscopic Nissen fundoplication between June 2004 and June 2021.

### 2.1. Analysed Outcomes

Following patient variables present at initial Nissen fundoplication were included in the study: gender, body weight, age at first fundoplication, preoperative gastrostomy placement, vomiting, hiatal hernia on initial upper gastrointestinal (UGI) contrast study or preoperative endoscopy, wrap failure and/or migration of the Nissen wrap on postoperative UGI study, respiratory symptoms, recurrent pneumonia, acute life-threatening events (ALTE) and neurological impairment. Surgical parameters included were operative approach, postoperative complications necessitating early intervention, and intraoperative findings during redo-fundoplications. Children who underwent a laparoscopic Nissen fundoplication without gastrostomy were compared to those with a pre-existing or simultaneously performed gastrostomy during initial fundoplication to identify a gastrostomy as a possible factor associated with redo fundoplication in NI children.

### 2.2. Study Population

Gastroesophageal reflux disease in all cases was diagnosed based on clinical history and documented by at least one of the following exams: UGI contrast study, endoscopy and/or 24-h pH study.

Esophageal pH and multichannel intraluminal impedance measurements are useful diagnostic tools in adults and older children, but are poor indicators of pathologic reflux in infants and do not adequately discern which patients will benefit from fundoplication [11]. In line with other centers, we predominantly focus on the patient’s clinical symptoms especially in neurologically impaired children to determine the need for fundoplication.

Postoperatively, feedings were initiated either by mouth or gastrostomy the day after the operation. In cases with simultaneous gastrostomy placement, feedings were started on the first postoperative day according to gastric residuals.

Our institutional postoperative standard protocol for NI patients who underwent laparoscopic fundoplication included an elective UGI contrast study for early follow up within the first postoperative month. This UGI contrast study should be performed prior to hospital discharge or transfer to the referring hospital. If a patient was discharged on weekends, the UGI study was postponed to the following week. Postoperative UGI studies were done in all patients through existing gastrostomies irrespective of their amounts of enteral feedings to minimize the possible risk of aspiration and to ensure adequate contrast volume administration. In patients without gastrostomies, contrast was given orally.

### 2.3. Laparoscopic Technique

All procedures were performed under general anesthesia with the patient in dorsal decubitus. A four or five trocar technique was used. At the beginning of the surgical procedure the correct position of the gastrostomy was ensured. The gastrostomy was re-inserted in cases when placed too close at the costal margin.

The Nissen fundoplication was performed using three non-absorbable sutures. The superior suture included the anterior aspect of the esophagus at the 11 o’clock position. After completion of the fundoplication, a gastrostomy was placed under laparoscopic guidance whenever needed.

In redo fundoplications the positioning of the trocars were like the initial operation. The crura were re-approximated posteriorly with two to three non-absorbable sutures with an appropriate esophageal dilatator in place. Mesh was not placed in this series. A 360° fundoplication was recreated using two to three non-absorbable sutures which incorporated the anterior aspect of the esophagus. In cases of a partial wrap breakdown, the re-fundoplication was recreated without complete takedown of the wrap when feasible.

### 2.4. Statistical Analysis

Patients’ data were presented with descriptive statistics including frequencies, percentages, means and ranges (as data were considered not normally distributed). Categoric data were compared using chi-square analysis. A *p* value < 0.05 was considered statistically significant.

## 3. Results

### 3.1. Overall Population

In total, 145 children underwent anti-reflux surgery during the study period. Patients who were operated on via laparotomy (*n* = 24), or underwent an anti-reflux procedure other than a complete Nissen wrap (*n* = 8), were excluded from the study. Furthermore, children with laparoscopic fundoplication without neurologic impairment (*n* = 10) were also excluded from the study. We included 103 patients, of which 60 were boys (58.3%). The patient demographics, follow up, preoperative complaints and preoperative diagnostics are displayed in Table 1. Causes for neurological impairment and number of patients with redo fundoplications are listed in Table 2.

A gastrostomy was placed during initial fundoplication in 41 patients (40%). Eleven patients (11%) received a concomitant revision of their gastrostomy during initial fundoplication. Thirty-nine (38%) patients had a gastrostomy prior to fundoplication.

The conversion rate to open surgery in the initial fundoplication series was 5% (5 in 103 patients). The reasons for conversion to open surgery were difficult visualization of the anatomic structures (*n* = 2), extensive adhesions (*n* = 1), instability during anesthesia (*n* = 1), and extreme scoliosis (*n* = 1). Since 2009, only one conversion had to be done due to extreme scoliosis.

### 3.2. Postoperative Examinations

A routine postoperative UGI contrast study was carried out in 94 of 103 patients (91%). In 83 patients (89%) the UGI contrast studies were performed within the first week following initial laparoscopic Nissen fundoplication. Except for one patient all postoperative UGI studies were carried out within the first postoperative month. 

Contrast was administered via gastrostomy in 77 patients (82%). Postoperative UGI studies via gastrostomy were carried out without any adverse events. In all cases, an appropriate amount of contrast was applied to evaluate the position and the integrity of the Nissen wrap. In 17 patients (18%), contrast was given orally. We did not experience aspiration pneumonias as adverse events following early postoperative UGI studies. In three of 17 patients with oral administration of contrast we documented an increased risk for aspiration. Nevertheless, the correct position of the wrap and sufficient flow of contrast into the stomach were confirmed in all patients.

Patients were followed periodically in the outpatient clinic after hospital discharge. Patients did not undergo 24-h pH studies monitoring to investigate the incidence of postoperative GER but were instead followed clinically. Indications for further endoscopy and/or UGI contrast imaging were clinical signs of recurrent gastroesophageal reflux.

Failure of the anti-reflux surgery was suspected when a patient was unable to gain weight and/or the re-occurrence of airway infections because of clinically obvious postoperative gastroesophageal reflux while receiving gastric feedings that did not respond to postoperative medical interventions. Accordingly, 13 patients who received a re-fundoplication underwent additional UGI contrast studies (12 patients; 92%) and/or endoscopy (7 patients; 58%).

### 3.3. Redo-Operations

Among the 103 children who underwent laparoscopic Nissen fundoplication, we identified 13 patients (12.5%) who had to be re-operated during the follow up period. The demographics of children who underwent a redo fundoplication are shown in Table 3. Eleven of the thirteen patients (85%) underwent one redo fundoplication, and two children (15%) underwent two redo fundoplications.

We experienced one early postoperative complication which necessitated early re-fundoplication. In this case, a 11.8 year old boy with a pre-existing gastrostomy was scheduled for a UGI contrast study on postoperative day 5. At that time, he tolerated gastrostomy feedings well and showed no obvious signs of postoperative reflux. The UGI contrast study revealed an intrathoracal herniation of the Nissen wrap and a laparoscopic re-fundoplication and re-hiatoplasty was carried out on the following day. The further postoperative course was uneventful. The UGI study after the redo and the follow up after 2.1 years revealed no signs of recurrence. 

Two re-operated patients (15%) died, one after the first redo 4.5 years after initial fundoplication, another after a second redo 4.8 years after initial operation (Table 3). This was in both cases due to their medical condition and not related to the surgical procedures.

Ninety patients were assigned to the gastrostomy group: thirty-nine patients already had a pre-existing, forty-one patients underwent concomitant gastrostomy placement and eleven received a concomitant revision of their pre-existing gastrostomy during initial fundoplication. The non-gastrostomy group comprised of thirteen patients. 

Twelve of the thirteen patients (92%) with redo operations either had a pre-existing gastrostomy (*n* = 5), simultaneously received a gastrostomy (*n* = 5), or their existing gastrostomy had to be replaced (*n* = 2) during initial laparoscopic fundoplication. 

We identified only one patient with redo- fundoplication in the non-gastrostomy group (1 out of 13; 8%) compared with twelve patients with redo fundoplications in the gastrostomy group (12 out of 90; 13%). However, the comparison of redo operations in both groups did not reach statistical significance (*p* = 0.607).

## 4. Discussion

The recognition of complications in the early postoperative period is mandatory. The treatment of early complications is often more straight forward and does not necessarily prolong hospital stay. Up to date there is no generally accepted algorithm for early postoperative follow-up after pediatric fundoplication available, especially information on NI children following laparoscopic fundoplication is lacking. Particularly with NI children the assessment of abdominal pain and other symptoms suggestive for GER can be challenging, especially in the early postoperative period. With pain being one of the most frequent signs of recurrent reflux (besides vomiting or regurgitation as obvious signs for GER), the early postoperative follow-up in NI patients has its pitfalls. Thus, it was our practice to perform a scheduled UGI contrast study within the first postoperative days to evaluate early postoperative outcome. Early postoperative follow-up of patients following fundoplication varies across practices and is frequently not reported in pediatric literature. Different strategies can be deduced from published data in different study groups. Lopez et al. [12] reported their case series of 417 patients (NI children 149; 36%) with laparoscopic fundoplication in which they performed elective UGI contrast studies one month after surgery. Celik et al. [13] mention that postoperative contrast studies were done selectively in 9 of their 72 children. In a prospective cohort study by Knatten et al. [14], the authors scheduled the first UGI contrast study six months after fundoplication. 

In our series of 103 patients, postoperative UGI studies were carried out in 65 children within the first five postoperative days (63% of all cases). If the patients were discharged on weekends, the postoperative UGI study was postponed to the following week or to the next outpatient appointment. Nevertheless, all but one were performed within the first postoperative month. 

However, in the present study we were able to identify early postoperative complications necessitating re-operation in only 1% of our cases. In contrast, 12 patients requiring redo fundoplication due to wrap failure and/or intrathoracal wrap herniation did not show any pathology at early UGI contrast studies. 

Kvello et al. [7] very recently reported 28 children with redo Nissen fundoplications. One patient presented with severe postoperative pain due to a herniated Nissen wrap on postoperative day one, and underwent re-fundoplication on postoperative day two. In addition, a retrospective study reported three early wrap herniations on the first postoperative day in 106 children following laparoscopic Nissen fundoplications [8]. Two wrap herniations appeared in NI children, and one in a neurologically normal child. In all three patients the intrathoracal wrap herniation was detected on a UGI contrast study on postoperative day one, and all three underwent redo surgery.

In accordance with our patient, all the reported children underwent immediate reoperations. In contrast to the one case reported by Kvello et al. [7], our patient did not show any clinical signs of reflux or abdominal compromise and might have been undiagnosed without the scheduled UGI contrast study. Unfortunately, Mathei et al. [8] did not report on the clinical symptoms in their three patients. Defining the criteria for the selective use of contrast studies is difficult, because the symptoms are highly subjective, and their assessment may vary between clinicians. However, except for one patient who underwent an early redo fundoplication on postoperative day six due to a herniated wrap, we did not identify any other early postoperative complications which needed further interventions. We therefore changed our institutional postoperative algorithm and no longer perform scheduled early postoperative UGI studies in NI patients, unless there are clear symptoms suggestive of postoperative GER.

The incidence of redo fundoplications in the present study is in line with previously published data. In a large multicenter study, the incidence of redo Nissen fundoplication was 12.2% and 21.6% in those with complete follow-up. The mean time to first redo fundoplication was 27.6 months [15]. A prospective study reported herniation of the wrap in 19.6% of NI children (9 of 46) compared with 4.8% (2 of 41 patients) in non-NI children [14]. 

In our patients collective, two out of thirteen patients (15%) required a second redo-operation. The time between initial fundoplication and first redo was 196 and 140 days, respectively. These findings are in line with Desai et al. [16], who postulated an increased likelihood of failure necessitating a second redo in cases of a shorter time between initial fundoplication and redo surgery.

To refine surgical technique in laparoscopic Nissen fundoplication there is growing evidence that esophageal dissection and mobilization is associated with increased incidence of intrathoracal wrap migration [17,18]. Initial outcomes were identical with 15% herniation in both NI and non-NI patients. However, long term follow-up showed a rise of the incidence of wrap herniation of 36.5% in the patients who received extensive esophageal mobilization compared with 12.2% in the minimal mobilization group. The long-term follow-up of their study did not stratify in NI and non-NI patients [17]. In continuation with leaving the esophagogastric membrane intact, St. Peter et al. [19] demonstrated the absence of wrap herniation after one year follow-up in 120 patients. Although the authors did not stratify in NI and non-NI patients, over 70% of their patients either had concomitant gastrostomy placement or had a gastrostomy prior to fundoplication. Another recent study further advocated the minimal dissection to prevent wrap transmigration in Nissen fundoplication [18]. Unfortunately, neurologic impairment was an exclusion-criteria in their study. Despite impressive short-term outcome data on wrap migration and warp failure with minimal esophagogastric dissection, the positive effect in NI children needs to be further elucidated.

Different reports examine factors associated with redo Nissen fundoplications in children. A typical combined procedure during laparoscopic fundoplication is the simultaneous placement of a gastrostomy, especially in NI children [20]. There is certainly a lack of data investigating a potential impact of pre-existing gastrostomy or concomitant placement of a gastrostomy during laparoscopic Nissen fundoplication in NI patients.

In our study, 12 out of 13 patients with redo operations had either an existing gastrostomy or underwent concomitant gastrostomy placement during initial fundoplication. A retrospective case series by Lopez et al. [12] reported a simultaneous gastrostomy placement in 88 of 149 NI children (59%) during initial laparoscopic fundoplication. Re-operations were done in 10 (7%) patients; with 7 of these patients having concomitant gastrostomy placement. Pre-existing gastrostomies at initial fundoplication were not quoted and not evaluated as a possible factor for redo operations.

In a prospective randomized trial which aimed to evaluate surgical technique to reduce the occurrence of postoperative wrap herniation, 17 patients had pre-existing gastrostomies and 72 patients underwent concomitant gastrostomy placement during initial fundoplications [19]. Patients were not stratified according to neurological impairment and redo operations were not evaluated according to simultaneous gastrostomy placement.

A very recent study reports on short and long term outcomes after pediatric redo fundoplications [7] in 24 patients. Most of these redo patients were neurologically impaired (16 of 24; 67%), a possible association with a pre-existing gastrostomy had not been reported.

Considering our observations, and inconsistent published data, a larger series is needed to investigate the potential association of gastrostomies with increased incidence of redo fundoplications especially after laparoscopic procedures in NI children.

## 5. Conclusions

In conclusion, the use of early upper gastrointestinal contrast imaging following anti-reflux surgery is not necessary as it does not identify a significantly greater number of acute postoperative complications requiring re-intervention in an otherwise asymptomatic patient.

We believe that the careful clinical assessment by an appropriate clinician with selective performance of a postoperative contrast study should be the preferred method in identifying early complications in NI patients. The impact of pre-existing or simultaneously placed gastrostomies during laparoscopic Nissen fundoplications need to be further investigated in a larger series.

## Figures and Tables

**Table 1:** Patient demographics, preoperative complaints, and preoperative diagnostics.

	Cohort
	(*n* = 103)
Age (years) at initial operation, mean	6.51 (0.11–18.41)
Body weight (kg) at initial operation, mean	16.22 (3.3–62.50)
Follow up (years), mean	4.15 (0.01–16.65)
Death, *n* (%)	16 (16%)
Average time (years) to death after initial fundoplication, mean	4.42 (0.25–12.45)
Preoperative Complaints	
Recurrent vomiting, *n* (%)	103 (100%)
Hematemesis, *n* (%)	15 (16%)
ALTE, *n* (%)	9 (9%)
Recurrent pneumonia, *n* (%)	83 (81%)
Respiratory infections w/o pneumonia, *n* (%)	13 (13%)
Respiratory infections and pneumonia, *n* (%)	24 (23%)
Preoperative Diagnostics	
Endoscopy preoperative, *n* (%)	76 (74%)
Esophagitis in endoscopy, *n* (%)	63 (83%)
Ulcerative esophagitis, *n* (%)	15 (20%)
Upper gastrointestinal contrast study, *n* (%)	76 (74%)
Reflux on UGI study, *n* (%)	63 (83%)
Hiatal hernia on UGI study, *n* (%)	10 (13%)
24-h pH study, *n* (%)	16 (16%)
24-h pH study pathologic, *n* (%)	14 (87%)

**Table 2:** Causes of neurological impairment and number of patients with redo fundoplication.

Causes of Neurological Impairment	*n* (%)	Patients with Redo Fundoplication (*n* = 13)
Perinatal anoxia injury/asphyxia	35 (34%)	4
Genetic disorder	22 (21.5%)	1
CNS anomalies	10 (9.7%)	3
Developmental delay of undetermined origin	10 (9.7%)	3
Metabolic disease	9 (8.7%)	1
Intraventricular hemorrhage	6 (5.8%)	0
CNS infections	3 (2.9%)	0
Postnatal hypoxic ischemic brain injury	3 (2.9%)	0
Seizure disorder	3 (2.9%)	0
Other	2 (1.9%)	1

**Table 3:** Demographics of patients undergoing redo fundoplication.

	Redo-Operations (*n* = 13)
First redo operation, *n*	*n* = 11
Age at primary fundoplication, mean (range)	9.1 years (0.9–14.9 years)
Age at redo fundoplication, mean (range)	11.6 years (2.8–16.76 years)
Time from primary fundoplication to redo, mean (range)	2.63 years (0.02–8.74 years)
Male, *n* (%)	5 (42%)
Redo- laparoscopic/open, *n*	11/1
Conversion to open, *n*	1
Wrap failure and hiatus intact, *n*	2
Wrap intact and wrap herniation, *n*	1
Wrap failure and herniation, *n*	8
Second redo operation, *n*	*n* = 2
Male, *n* (%)	0 (0%)
Age at primary fundoplication	4.3 and 5.4 years
Time from primary fundoplication to redo	196 and 140 days
Redo- laparoscopic/ open	2/0
Conversion to open	0/0
Wrap failure and wrap herniation, *n*	1
Wrap herniated, *n*	1
Time from first redo to second redo operation	348 and 167 days
Second Redo—laparoscopic/open	1/1
Conversion to open, *n*	0
Wrap failure and wrap herniation, *n*	1
Wrap failure and hiatus intact, *n*	1

## Data Availability

The data that support the findings of this study are available on request from the corresponding author. The data are not publicly available due to privacy or ethical restrictions.
